# Pathological Fracture on a Cyst-Like Mandibular Lesion Revealing a Primary Intraosseous Squamous Cell Carcinoma: A Rare Clinical Case and Review of the Literature

**DOI:** 10.7759/cureus.65967

**Published:** 2024-08-01

**Authors:** William Calvin, Nicolas Ullmann, Gaoussou Toure

**Affiliations:** 1 Maxillofacial Surgery, Centre Hospitalier Intercommunal de Villeneuve-Saint-Georges, Villeneuve-Saint-Georges, FRA

**Keywords:** pathological fracture, oral malignancy, odontogenic carcinoma, intraosseous neoplasm, primary intraosseous squamous cell carcinoma

## Abstract

Primary intraosseous squamous cell carcinoma (PIOSCC) is a rare malignant tumor originating from remnants of odontogenic epithelium within the central jaw bone. The majority of the PIOSCC cases are diagnosed incidentally on histological findings while a potential malignancy wasn’t necessarily determined at first on initial investigations. The diagnosis of PIOSCC is challenging and relies on ruling out other types of carcinomas, including metastatic tumors from other primary sites. It is essential for the physician to detect potential clinicoradiologic "red flags" for an early diagnosis and appropriate treatment. In this article, we present an unusual case of PIOSCC discovered on a cyst-like lesion with pathological fracture and review the current literature to identify the potential warning features.

## Introduction

Primary intraosseous squamous cell carcinoma (PIOSCC) is a seldom-discussed malignant tumor developing from the remnants of odontogenic epithelium and represents about 2% of all oral SCC cancers [1,2]. First documented by Loos in 1913 as a "central epidermoid carcinoma of the jaw", this condition is defined by the emergence of a malignant tumor within the jaw bone and with no initial connection with the overlying oral mucosa. Wills renamed it as "intra-alveolar epidermoid carcinoma" in 1948, which was later modified by Shear as "primary intra-alveolar epidermoid carcinoma". The World Health Organization (WHO) finally proposed the term "primary intraosseous squamous cell carcinoma" in 1972 and a subsequent classification into different subtypes whether the lesion originates from the remnants of the odontogenic epithelium (Malassez's epithelial rest) or from a preexisting benign lesion (odontogenic cyst or another tumor). Due to its rarity, establishing diagnosis is challenging because of the lack of pathognomonic findings and it also requires ruling out the possibility of a metastatic primary tumor at a distant site.

Previous studies attempted to report the clinicopathological features and management of this tumor in order to better understand the disease. However, the available data are very limited with approximately around 200 cases reported. This study explores the case of a 77-year-old male patient provisionally diagnosed with a pathological fracture resulting from a large odontogenic cyst, later identified as PIOSCC. It offers a review of the few existing cases described in the literature.

## Case presentation

In January 2023, a 77-year-old man was referred to the department of maxillo-facial surgery at Villeneuve-Saint-Georges Hospital in France with a history of a left mandibular fracture that occurred while eating 15 days ago. Prior to his visit, a cure of antibiotics (amoxicillin 3g per day for 10 days) had been prescribed by the surgeon who examined him in a private clinic. The patient’s medical history was otherwise unremarkable. In particular, no tobacco or alcohol misuse was reported.

**Figure 1:**
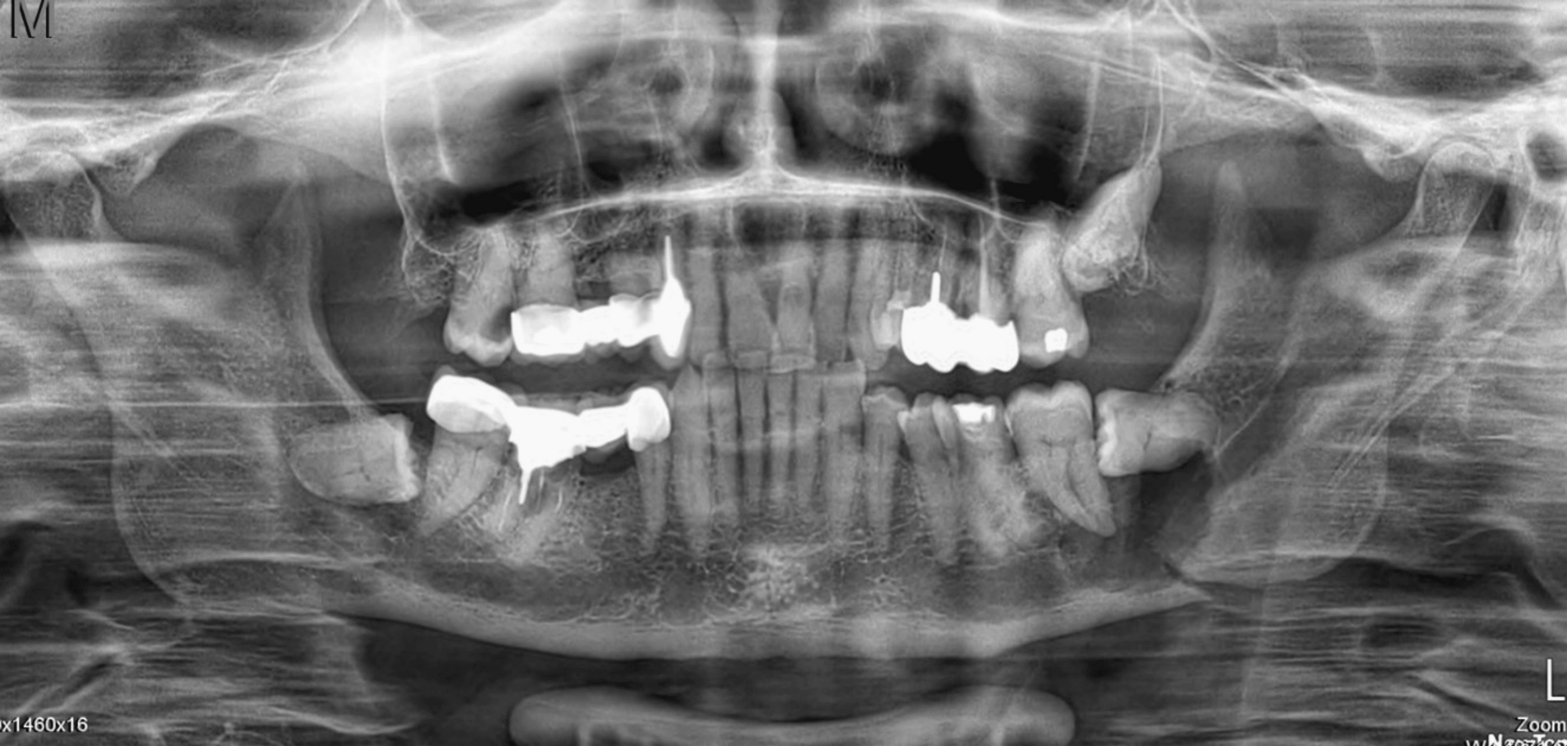
Pre-operative radiological findings A panoramic radiograph showing a cyst-like lesion with ill-defined borders that extended from the distal part of the third impacted molar to the mesial part of the second molar.

**Figure 2:**
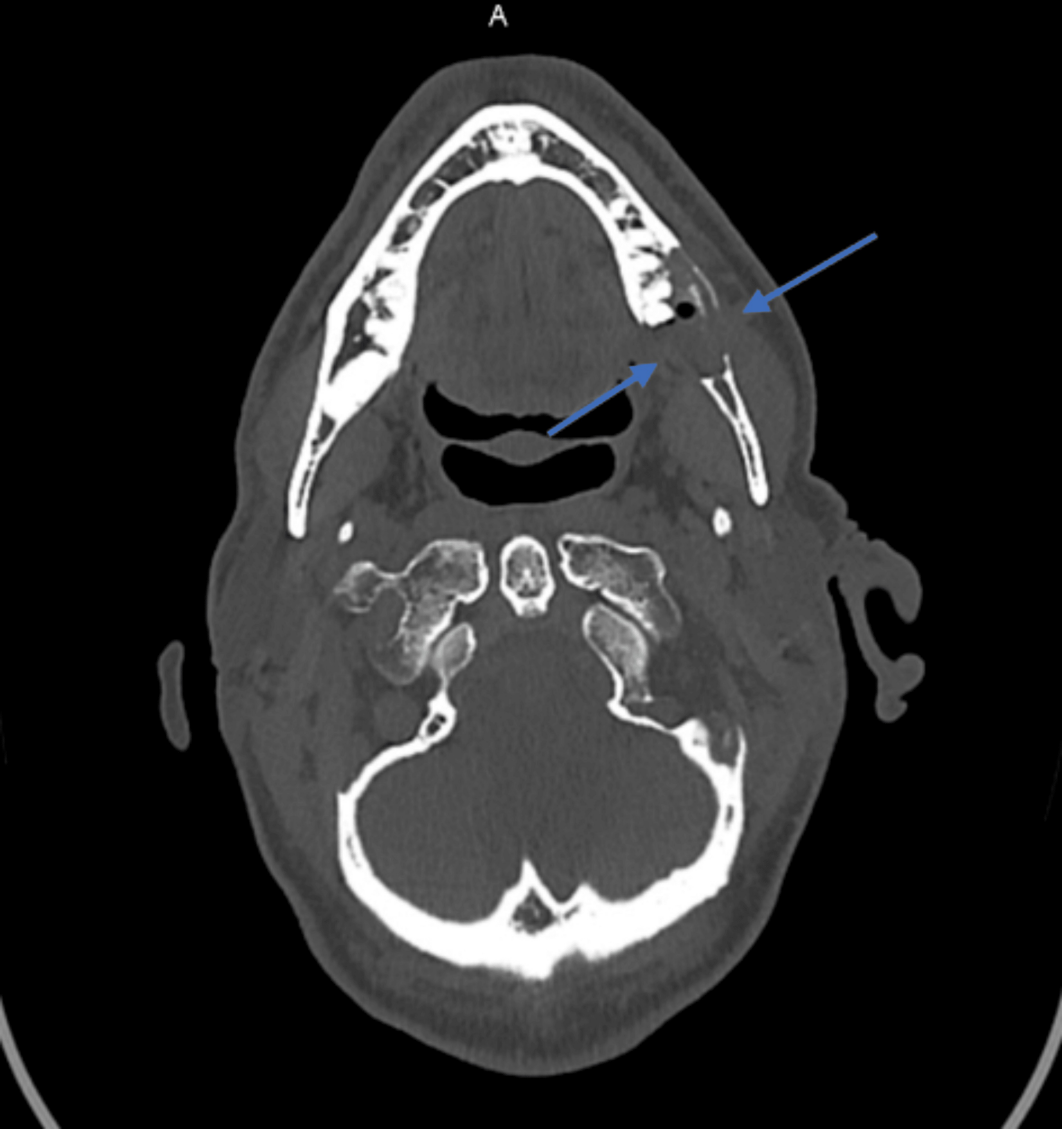
Pre-operative radiological findings Contrast-enhanced computed tomography showing an osteolytic lesion with invasion of the infra-alveolar nerve canal and bi-cortical perforation (blue arrows).

Extra-oral examination revealed painful trismus with 2.5 cm of mouth opening without any sensitive disorder (paresthesia/hyperesthesia) or swelling noticed in the left buccal region. No lymphadenopathy was palpable on submandibular and cervical examination. Intra-oral examination revealed signs of malocclusion with a deviated dental midline toward the fracture and an inability to bring the teeth together. No mucosal abnormalities were present on the buccal and lingual gingiva except for a slight erythema in the left retromolar region. The tooth n° 37 was painful on palpation with physiological mobility and no sign of pus discharge. A panoramic radiograph showed a cyst-like lesion with ill-defined borders that extended from the distal part of the third impacted molar to the mesial part of the second molar (Figure 1). The lesion was associated with a fracture of the left mandibular angle. No teeth resorption was observed and the infra-alveolar nerve canal seemed to be displaced on the lower part of the mandible. A pre-operative enhanced CT revealed a lesion of the size 28 x 15 mm invading the infra-alveolar nerve canal with perforations of the buccal and lingual cortical plates (Figure 2). 

**Figure 3:**
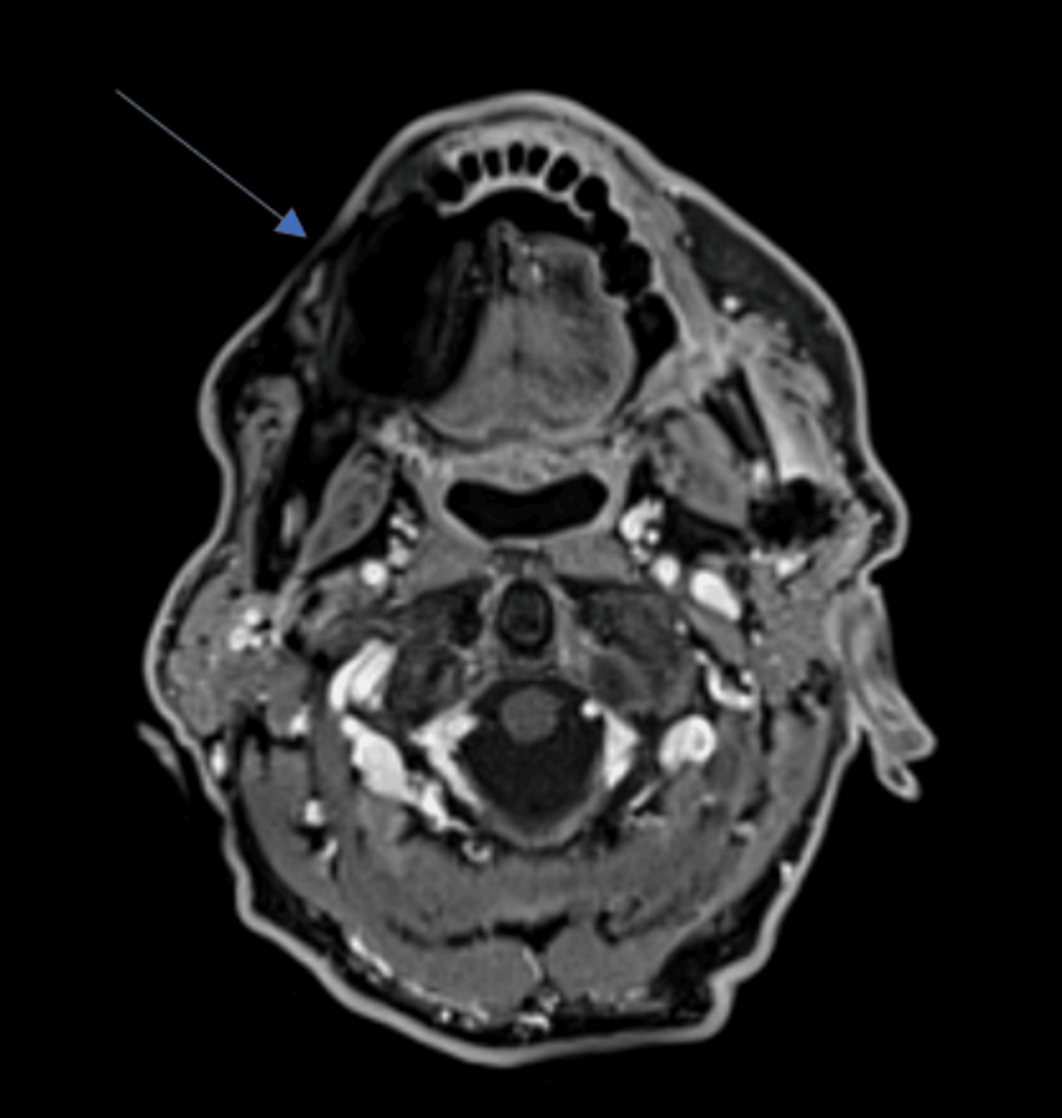
Assessment of the radiological extension Head and neck contrast-enhanced magnetic resonance imaging revealing an infiltration of the left mandible corpus with peripheral enhancement (blue arrow).

**Figure 4:**
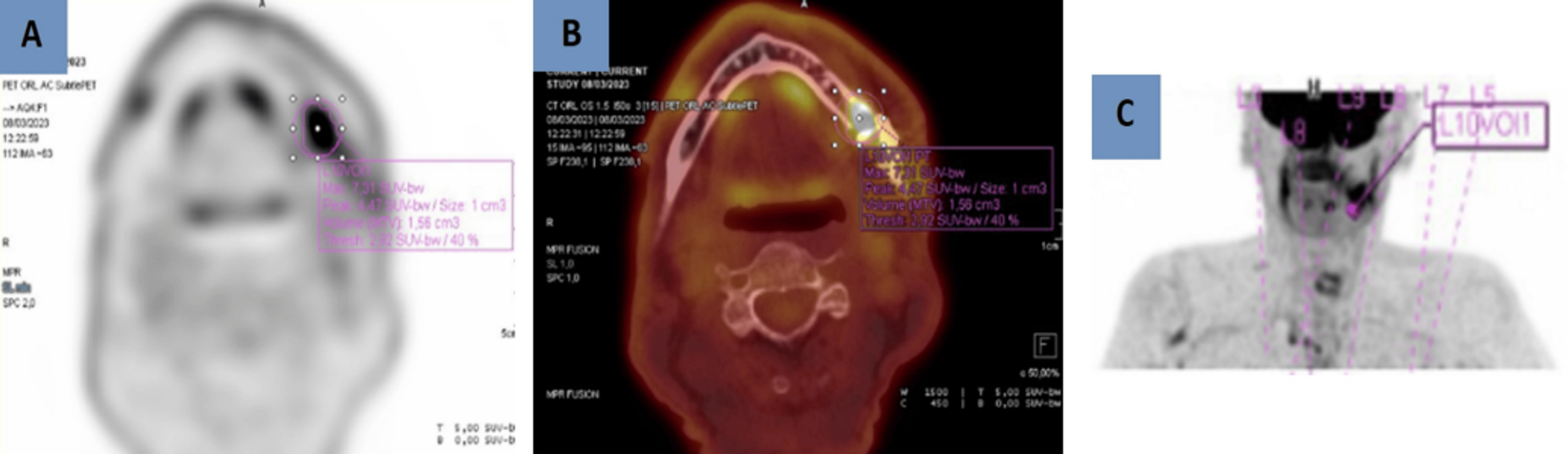
Assessment of the radiological extension Axial 18F-FDG PET (A), axial-fused PET/CT (B), and MIP (C) images showing intense focal focus (SUV max 7.3) corresponding to a lytic lesion located between the two screws anterior osteosynthesis material at the base of the ascending ramus of the left mandible, continuing toward the horizontal branch of the mandible. No laterocervical or supraclavicular lymph node fixation is detected. PET: positron emission tomography, CT: computed tomography, MIP: maximum intensity

Based on these findings, a provisional diagnosis of a dentigerous cyst with mandibular pathological fracture was made and surgical enucleation of the lesion was scheduled under general anesthesia with extraction of the left wisdom tooth and osteosynthesis using a titanium reconstruction plate. The specimen was sent for histopathological examination. The extirpated surgical specimen consisted of a set of six fragments measuring from 2.8 x 1 cm to 0.8 x 0.3 cm. Microscopic examination was consistent with a moderately differentiated intraosseous squamous cell carcinoma arising de novo with a strong P16 expression and poorly keratinized. In light of this diagnosis, further imaging was performed to exclude potential differential diagnosis and for the assessment of the tumor’s extension. Contrast-enhanced magnetic resonance imaging (MRI) revealed infiltration of the left horizontal branch of the mandible with peripheral enhancement and reactive thickening of the soft tissues (Figure 3). The CT of the head and neck and thoraco-abdominopelvic region showed no evidence of lymphadenopathy or distant metastasis. The positron emission tomography (PET) scan showed a hypermetabolic lesion with a lytic aspect in contact with the left mandibular osteosynthesis material with no argument for loco-regional or distant extension (Figure 4). A panendoscopy was also performed as a screening examination for simultaneous primary tumors, which showed no suspect lesion upon the upper aero-digestive tract. A percutaneous gastrostomy was also executed at the same time for postoperative nourishing conditions.

**Figure 5:**
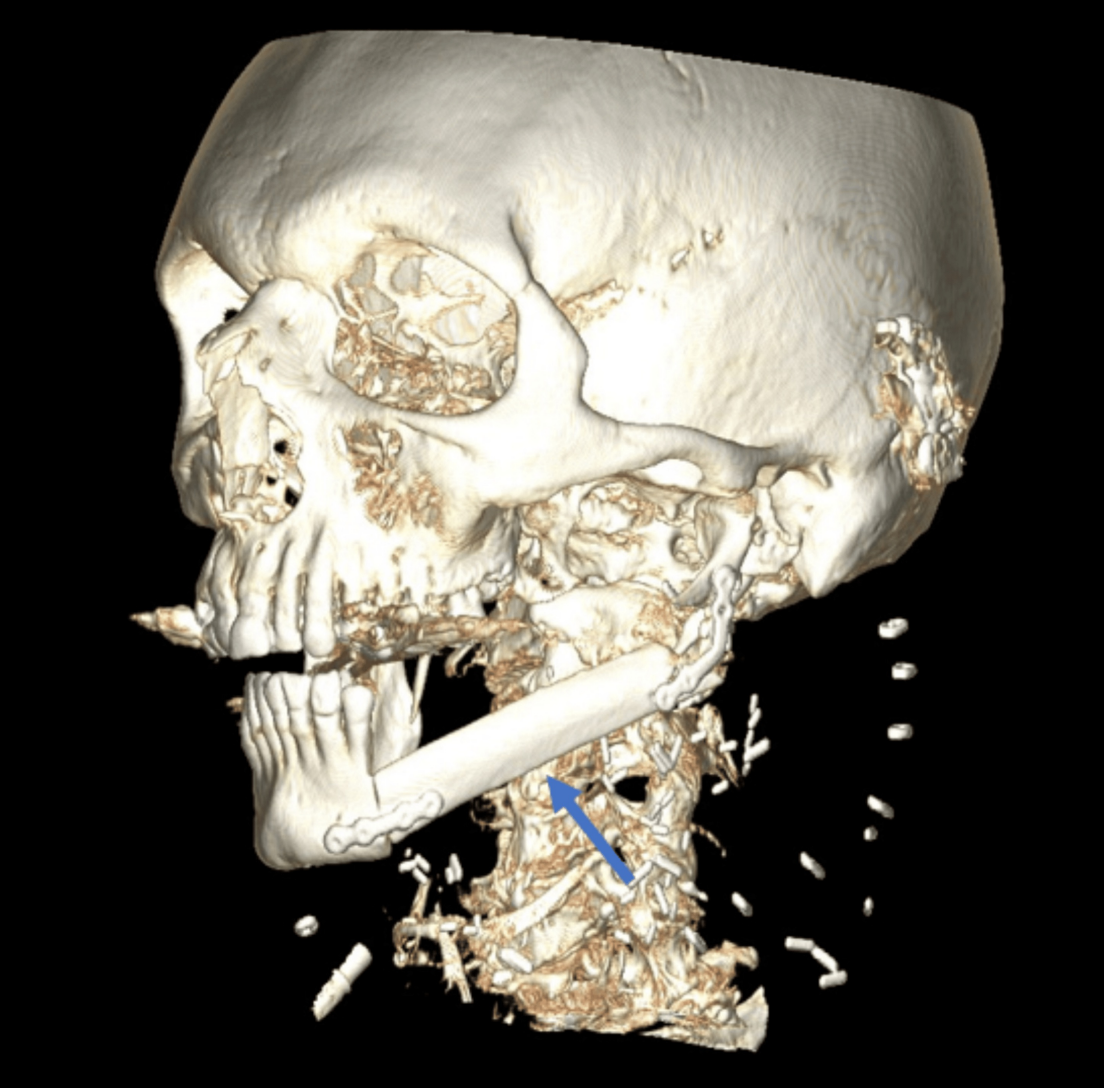
Postoperative three-dimensional scanner reconstruction imaging Fibular free flap reconstruction of the left mandibular defect (blue arrow).

**Figure 6:**
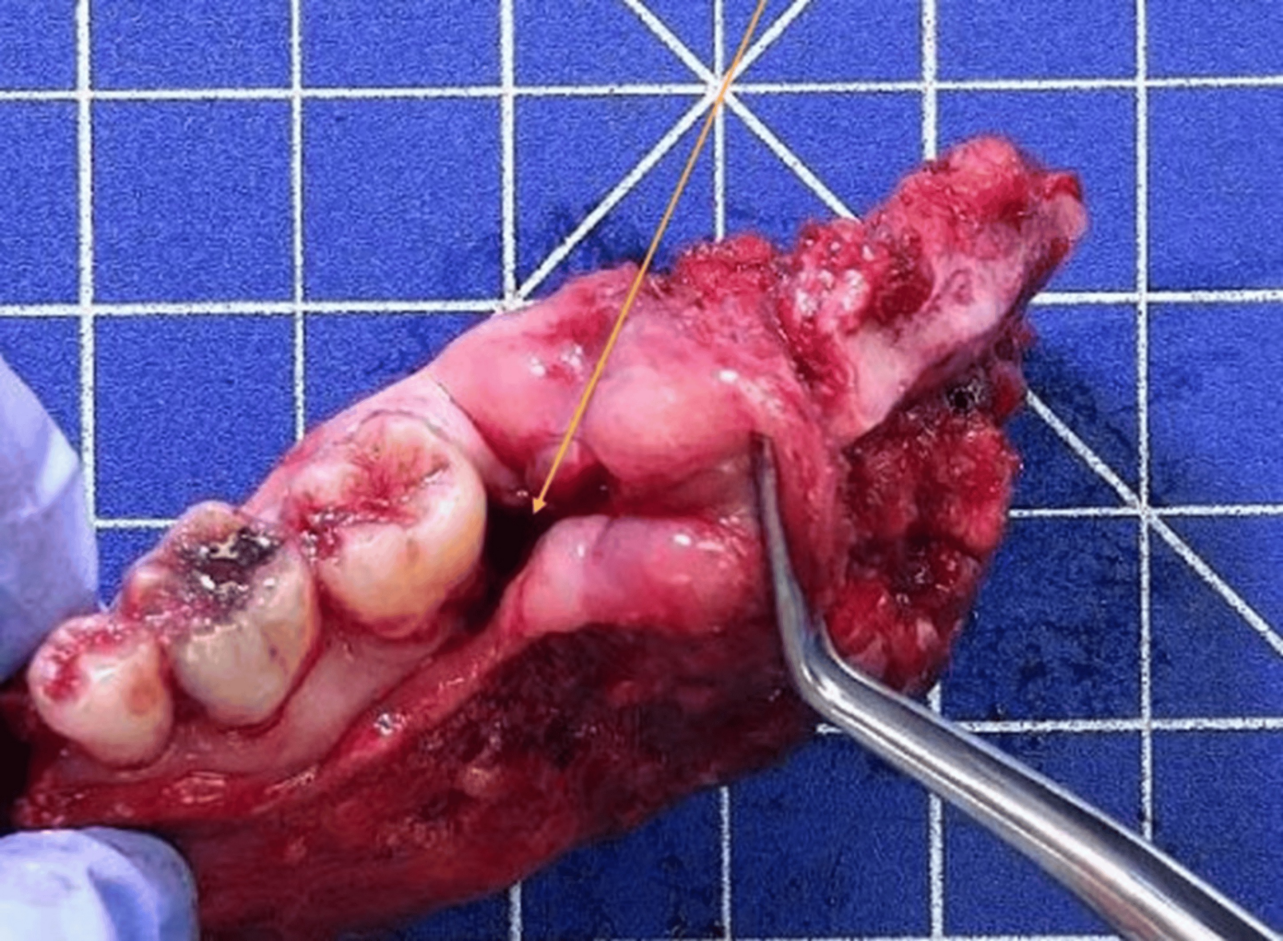
Histopathological findings of the surgical specimen Macroscopic examination of the resected left mandible sized 8 x 4.5 x 3 cm (yellow arrow).

**Figure 7:**
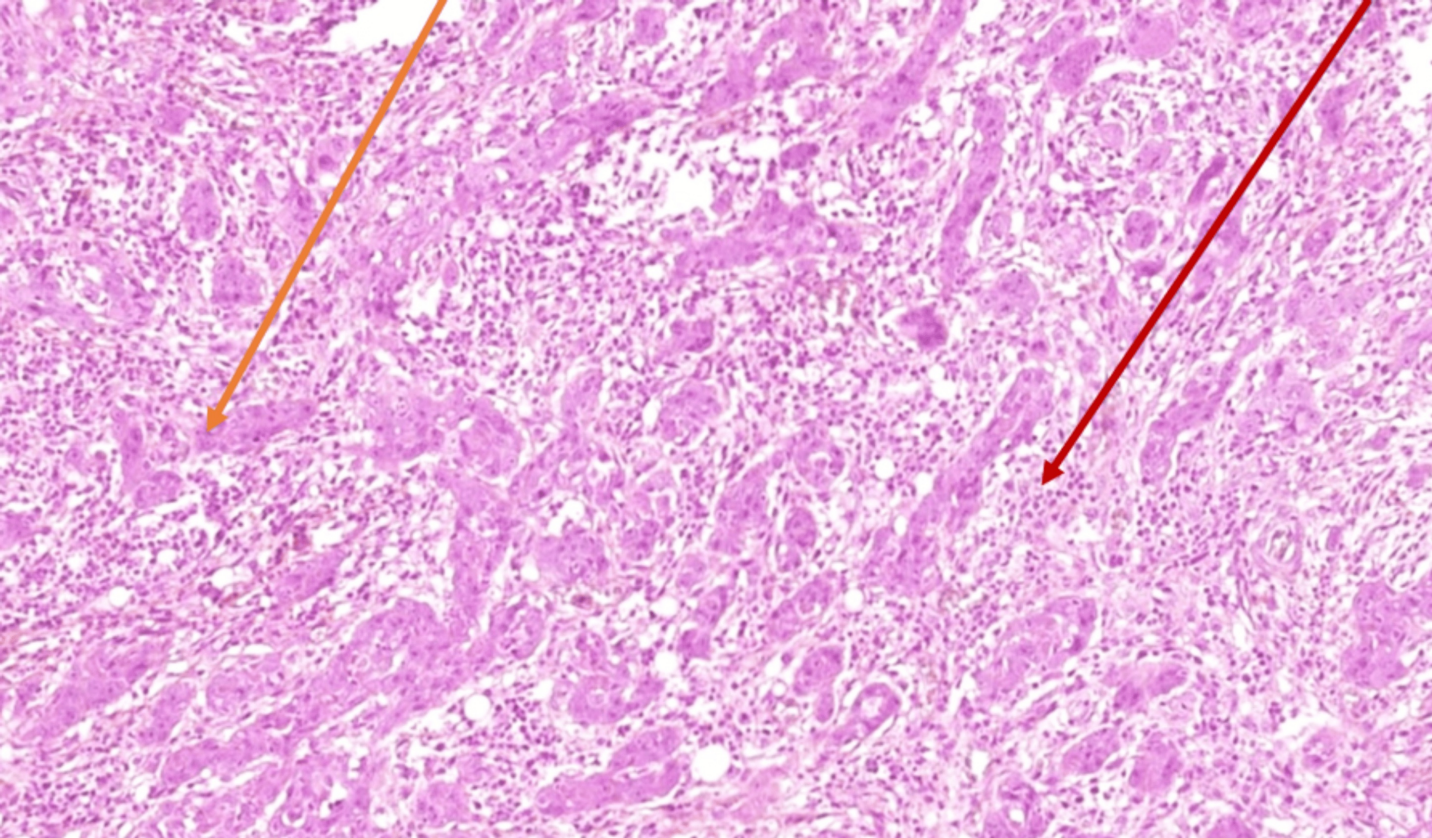
Histopathological findings of the surgical specimen Microscopic examination (hematoxylin and eosin (H&E) staining; magnification, 200x ; scale bar, 50µm) showing a fibro-inflammatory tissue (red arrow) comprising a tumor proliferation made of nests and masses of medium- to large-sized cells with a few foci of keratinization and a few cell union bridges. A multitude of cytonuclear atypia with atypical mitoses (orange arrow). This tumor proliferation invades the bone tissue with lots of bony spaces surrounded by tumor masses.

The results were presented in a multidisciplinary consultation meeting and a left interruptive mandibulectomy with homolateral neck dissection and reconstruction with a fibular free flap was proposed with postoperative radiotherapy (Figure 5). The lesion was considered stage IV because of the initial bone involvement. The patient underwent an intervention in March 2023: a surgical piece of 8 x 4.5 x 3 cm was resected and the results of the first biopsy were confirmed (Figures 6, 7). The definitive results consisted of an intraosseous squamous cell carcinoma with free surgical margins and no lymph node metastasis for a total of 46 lymph nodes removed.

During the postoperative phase, an episode of nosocomial pneumopathy with septic shock caused by *Morganella morganii* occurred 10 days after surgery and led the patient to intensive care. The infection gradually reduced under a high dose of antibiotics (Tazocillin and Amikacin) and the patient was discharged 27 days after surgery. He completed adjuvant radiotherapy with a total of 33 sessions, which ended in June 2023. Sixteen months after the surgery the patient remains free of disease.

## Discussion

PIOSCC is an uncommon form of oral cancer, which originates within the jawbone and does not initially involve the oral mucosa. Instead, it develops from residual odontogenic epithelium. The exact pathogenesis remains unknown and no risk factor has clearly been identified; especially alcohol and tobacco misuse seem not to be implied in contrast to classical oral squamous cell carcinoma. Nonetheless, evidence suggests that prolonged inflammation and/or infection may contribute to the development of SCC with prolonged exposure to chronic inflammation, which represents a trigger for malignant transformation of the epithelium [3, 4].

To meet the specific criteria for diagnosis, several conditions must be satisfied. These include the absence of oral ulceration or any communication with the surrounding mucosa, the absence of an unrelated primary tumor at the time of diagnosis, and histologic evidence of the presence of squamous cell carcinoma. WHO proposed a recent classification with different subtypes depending on the origins of the odontogenic epithelium: PIOSCC arising de novo (type 1), PIOSCC originating from the lining of an odontogenic cyst (type 2), or from a benign odontogenic tumor such as a keratocyst or an ameloblastoma (type 3) [5]. Intraosseous carcinomas, in most cases, originate from the epithelial lining of odontogenic cysts, particularly from radicular, residual, and dentigerous cysts.

The clinical presentation is unspecific, but some variables were more frequent, which makes it possible to identify potential clinical "red flags", which should draw the attention of the practitioner: a middle-aged male (average age of 69.3 years old) who complains about a mandibular painful swelling and/or numbness or hypoesthesia and tooth loss [6,7]. History of tooth extraction is also frequently reported, which raises some questions about the physiopathology of the lesion: does the tumor originate from the remnants of post-extractionnal tissues or was the tumor causing tooth mobility that later motivated the extraction? Actually, focusing on just a specific population is risky as it can lead to missed diagnosis: as highlighted by Hyun Jun Oh et al., 12 cases already occurred in the pediatric population (ages ranged from 4 to 18 years) [8]. Moreover, a significant number of patients are completely asymptomatic or present only discrete symptoms and the lesion is revealed on a routine radiopanoramic [7]. Therefore, physicians should proceed carefully and particular attention should be paid on radiologic findings. However, PIOSCC often masquerades as a common odontogenic cyst due to its various presentations, which often leads to a delay in diagnosis and contributes to the unfavorable prognosis associated with this tumor. The survival rate at two and five years was 68.9% and 38.8%, respectively [9]. In a recent publication, Garzino-Demo et al. tried to identify the clinical and radiological characteristics of SCC arising from an odontogenic cyst: the lesion often presented as an osteolytic mass with diffuse cortical discontinuity, intense periosteum reaction, peripheral remineralization and dislocation of the inferior alveolar nerve (IAN) [10]. One other striking element is the absence of root resorption, classically expected in other benign lesions, with a typical "floating teeth" appearance implying the fast expansion of the lesion [11]. In our case, the clinical presentation was dominated by the pathological fracture, which explained the pain and the limitation of the mouth opening, but after a retrospective review, some radiologic features were suspected because of the absence of cortical expansion and tooth resorption, which is quite unusual for a benign cyst and the irregular borders. In consequence, despite having any pathognomical pattern, meeting some of those clinical and radiological records should prompt a more cautious approach, leading to an incisional biopsy to obtain a definitive histologic diagnosis every time there is an incertitude. Identifying those characteristics is, in fact, essential for a prompt diagnosis and an effective treatment.

The prognosis for PIOSCC is generally unfavorable, characterized by low survival rates and a tendency for recurrence and metastatic localization. In fact, a significant number of patients were poorly treated because the initial diagnosis was incorrect. Positive node metastases serve as a significant prognostic indicator for PIOSCC, along with a high histological grade and advanced N classification, all of which are associated with poorer outcomes (9). Additionally, cases of SCC originating from cysts tend to have a more favorable prognosis compared to those arising de novo [12].

## Conclusions

Primary intraosseous squamous cell carcinoma often presents some radiological features including osteolytic bone erosion with cortical perforation, periosteum reaction, infiltration of the inferior alveolar nerve, and a floating teeth appearance, which should lead to a careful diagnostic approach when detected. Thus, a comprehensive radiological evaluation is imperative to reduce the risk of misdiagnosis and to ensure the implementation of appropriate treatment strategies. Early diagnosis is indeed a key to a better prognosis of this pathology.
